# Genotyping and clonal origin of *Sporothrix brasiliensis* in human sporotrichosis cases in Argentina

**DOI:** 10.1016/j.mmcr.2024.100633

**Published:** 2024-02-16

**Authors:** Norma B. Fernandez, Bram Spruijtenburg, Iris N. Tiraboschi, Jacques F. Meis, Ana Lugo, María Cecilia López Joffre, Eelco F.J. Meijer

**Affiliations:** a Sección Micología, División Infectología, Hospital de Clínicas “José de San Martín”, Universidad de Buenos Aires, Av. Córdoba 2351, Ciudad Autónoma de Buenos Aires, C1121ABJ, Argentina; b Radboudumc-CWZ Center of Expertise for Mycology, Nijmegen, the Netherlands; c Canisius-Wilhelmina Hospital (CWZ)/Dicoon, Nijmegen, the Netherlands; d Institute of Translational Research, Cologne Excellence Cluster on Cellular Stress Response in Aging-Associated Diseases (CECAD), Excellence Center for Medical Mycology (ECMM), University of Cologne, Cologne, Germany; e Departamento Micología, Instituto Nacional de Enfermedades Infecciosas INEI-ANLIS “Dr. Carlos G. Malbrán” Av. VélezSarsfield563, Ciudad Autónoma de Buenos Aires, C1282AFF, Argentina; f División Infectología, Hospital de Clínicas “José de San Martín”, Universidad de Buenos Aires, Av. Córdoba 2351, Ciudad Autónoma de Buenos Aires, C1121ABJ, Argentina

**Keywords:** *Sporothrix brasiliensis*, Sporotrichosis, Zoonosis, Genotyping, Argentina

## Abstract

*Sporothrix brasiliensis* is considered a highly virulent emerging pathogen that causes sporotrichosis in humans, mainly after zoonotic transmission from infected cats. The epidemic of this zoonosis that originated from Brazil has spread in the last decades, generating hyperendemic regions in Latin America. We present two cases of human sporotrichosis causes by *S. brasiliensis* in Buenos Aires, Argentina, with good clinical response to differing treatments after contact with sick cats. Using Short tandem repeat (STR) genotyping, the two *S. brasiliensis* cases appear to be introduced from Brazil and likely originate from the same source.

## 1 Introduction

Sporotrichosis is currently the most prevalent and globally distributed implantation mycosis with regions of high endemicity caused by *Sporothrix* species. Within the clinically relevant clade of this thermodimorphic endemic fungus, *Sporothrix brasiliensis* stands out as the most virulent species [1]. This species has generated a zoonotic epidemic in Brazil since the 1990s that spread during the last decade to most Brazilian states, and to Latin American countries such as Argentina, Chile, Paraguay, and recently to the United Kingdom [[1], [2], [3], [4], [5], [6]]. *S. brasiliensis* is transmitted in the yeast phase by implantation through scratching, biting, direct contact with feline exudative mucocutaneous lesions or respiratory droplets expelled by infected or colonized domestic cats (*Felis catus*) [7]. A cat infected by *S. brasiliensis* often remains symptomatic, and may develop multiple cutaneous lesions with subsequent dissemination, often resulting in death in the absence of treatment [1]. With Short tandem repeat (STR) genotyping, it was shown that most present day cases in Brazil allocate to a widespread clade and that clonal transmission is frequent [2]. However, genotyping of Argentinian strains has not been performed to date. It is unknown whether *S. brasiliensis* cases in Argentina are imported from Brazil or introduced locally from the environment.

Here, we present two cases of human sporotrichosis caused by *S. brasiliensis* in Buenos Aires, Argentina, with a good clinical course. STR genotyping indicated that both cases share a common source, likely introduced from Brazil.

## 2 Clinical cases

### 2.1 Case 1

**Fig. 1:**
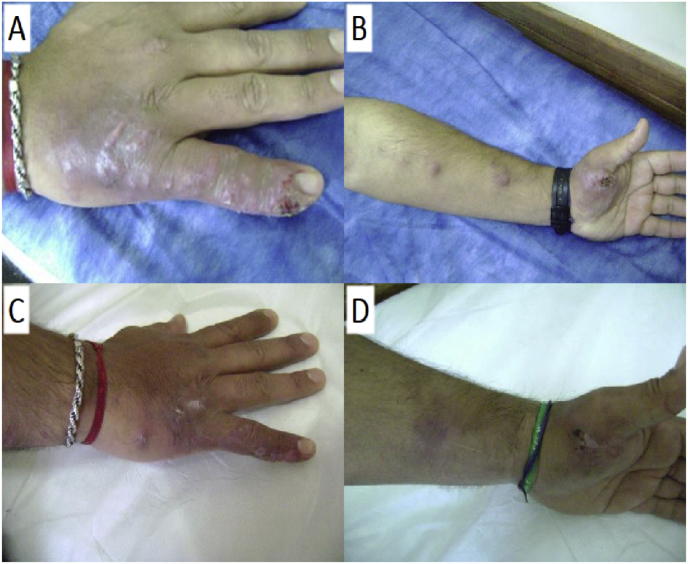
Clinical image of case 1 before (A/B) and after treatment (C/D).

A 35-year-old man living in the town of San Miguel, in the north of Buenos Aires province, Argentina, consulted for lesions on both hands and upper limbs in June 2014 (day 0). He had no previous history of disease or immunosuppression. The man reported that the lesions started 1 month earlier (day - 30) after being scratched and bitten while treating his sick cat, which had ulcerated lesions in the cephalic region. He received various antibiotic treatments in consultations with other medical institutions (amoxicillin-clavulanic acid, trimethoprim-sulfamethoxazole and rifampicin) without clinical improvement. Clinical examination on day 0 found two erythematous plaques with erosive-crusted lesions in their center, slightly painful on both hands (Fig. 1A and B). Both plaques appeared simultaneously, followed by multiple erythematous nodules of hard elastic consistency of different sizes (1–5 cm) following the lymphatic pathway towards the proximal region of both upper limbs and palpable right axillary nodes. Blood results demonstrated elevated liver enzymes (AST 133 IU/ml, ALT 197 IU/ml, ALP 206 IU/l), alteration of cholesterol and triglycerides (cholesterol 233 mg/dl, triglycerides 410 mg/dl). Abdominal ultrasound showed no abnormalities. Skin puncture of the lesion on the hands and one of the patient's forearm nodules was performed. Direct microscopic examination (fresh examination, with KOH 40%, Giemsa) of both materials was negative. *Sporothrix* spp. was isolated at 28 °C on Sabouraud dextrose agar (day 13), and subsequently identified by partial Intertranscribed spacer (ITS)1–2 and calmodulin (CaM) sequencing as *S. brasiliensis* [8]. Treatment was started with saturated solution of potassium iodide (SSKI) 5 drops every 8 h orally, increasing the dose daily up to 40 drops 3 times a day (day 15). The treatment was tolerated well and he was discharged after 4 months of treatment (day 120) (Fig. 1C and D).

### 2.2 Case 2

**Fig. 2:**
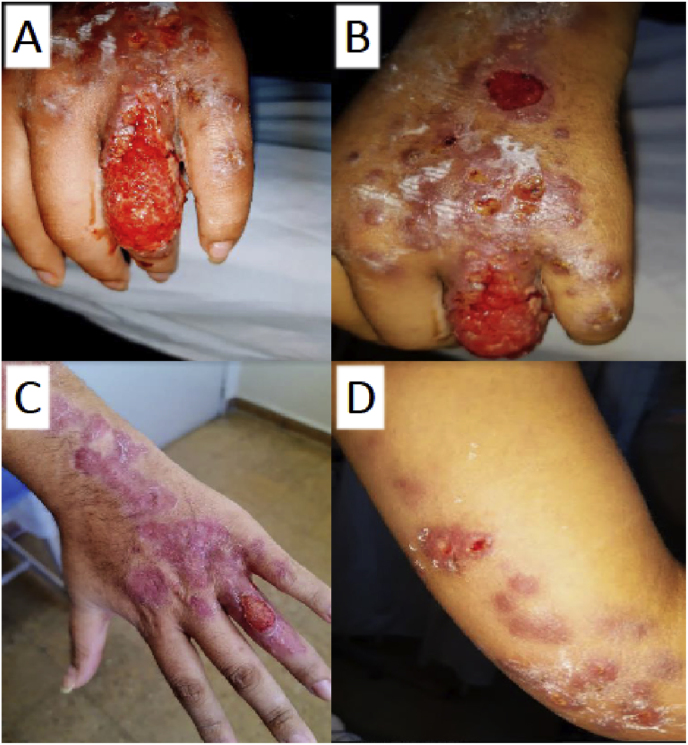
Clinical image of case 2 before (A/B) and after treatment (C/D).

**Fig. 3:**
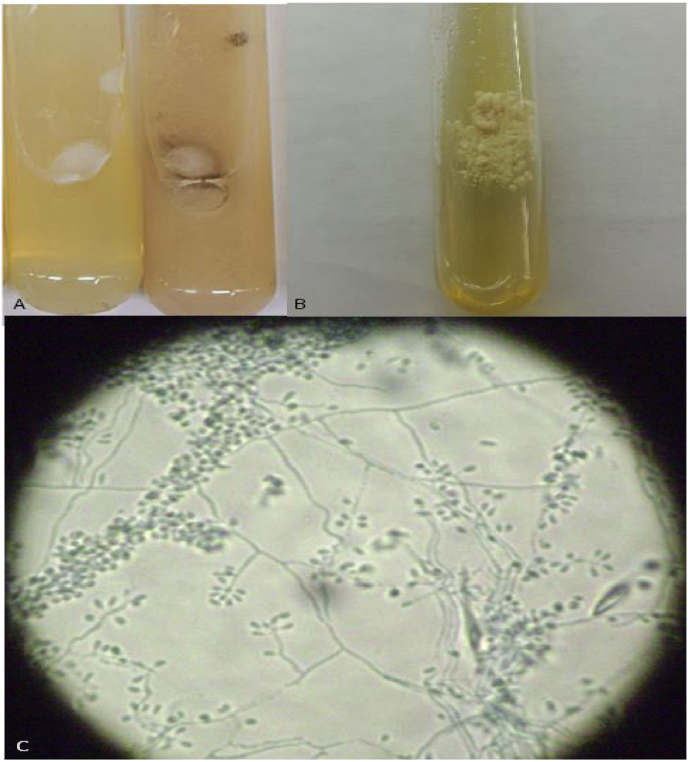
*S. brasiliensis* (A) Colonies at 28 °C on SDA (Sabouraud dextrose agar, left) and Lactrimel agar (right) (B) Colonies at 35 °C on SDA (C) Microscopy of mycelium form: hyaline hyphae, septate, fine, branched, with small conidia in sympodial arrangement (Lactophenol cotton blue 400X).

A 21-year-old woman living in Del Viso, north of Buenos Aires province, was admitted to the hospital in June 2020 for presenting with panniculitis, with multiple nodular lesions on the left upper limb (day 0). The patient had no relevant history, was in good general condition, with no other systemic symptoms and no contact with plants or recent trauma. History taking revealed her having had several medical consultations in other health institutions, which indicated antibiotic treatment (fusidic acid, cephalothin) without clinical improvement. The lesions started 3 months ago on the hand and progressed to the forearm (day −90). The patient reported contact with her sick cat which exhibited lesions in the cephalic region. The patient presented multiple painful erythematous nodules (about 20) of hard elastic consistency of different sizes (1–10 cm), some erosive and ulcerated, following the lymphatic pathway to the proximal region of the left upper limb, without palpable axillary adenopathy (day 0) (Fig. 2). A verrucous erosive plaque on the dorsal aspect of the second and third phalanx of the fourth finger and other erosive nodular lesions on the third phalanx of the fifth finger of the left hand (day 0). The patient had a normal hepatogram. Direct examination (fresh examination, PAS, Giemsa and Ziehl Neelsen staining) and culture for bacteria, mycobacteria from skin puncture and nodule biopsy were negative. After 12 days of incubation of both samples at 28 °C on Sabouraud dextrose agar, colonies of *Sporothrix* spp. were identified (day 12). The isolate was identified by transformation of the mycelial phase to yeast at 37 °C (Fig. 3) and partial sequencing of the CaM gene identified *S. brasiliensis*. She received itraconazole 400 mg/d (tablet) for 12 weeks with treatment success. She did not return after that time.

Generated calmodulin sequences in the current study were deposited under Genbank accession numbers PP080169 and PP080170. This work has the approval of the Bioethics Committee of the Hospital de Clinicas UBA for publication.

Genotyping of both isolates was performed using Short tandem repeat (STR). DNA was extracted and purified as described before [9]. In short, isolates were lysed using the MagNA Lyser system (Roche Diagnostics GmbH, Mannheim, Germany) and nucleic acids were subsequently isolated with the MagNA Pure 96 instrument, MagNA Pure and Viral NA Small Volume Kit and the Pathogen 200 SV protocol (All Roche Diagnostics GmbH), following the manufacturer's instructions. Multiplex PCR was performed as previously described [2]. Briefly, multiplex PCR was performed using 1x FastStart Taq buffer, deoxynucleotide triphosphates (dNTPs) (0.2 mM), MgCl2 (3 mM), forward and reserve primers (1–8 μM), 1 U FastStart Taq polymerase (Roche Diagnostics GmbH). PCR products were diluted 1:1000 in water, and 10 μL together with 0.12 μl Orange 600 DNA size standard (NimaGen, Nijmegen, The Netherlands) were incubated for 1 min at 95 °C and analyzed on a 3500 XL genetic analyzer (Applied Biosystems, Foster City, CA, USA). Copy numbers of the current isolates were compared to earlier typed isolates [2].

**Fig. 4:**
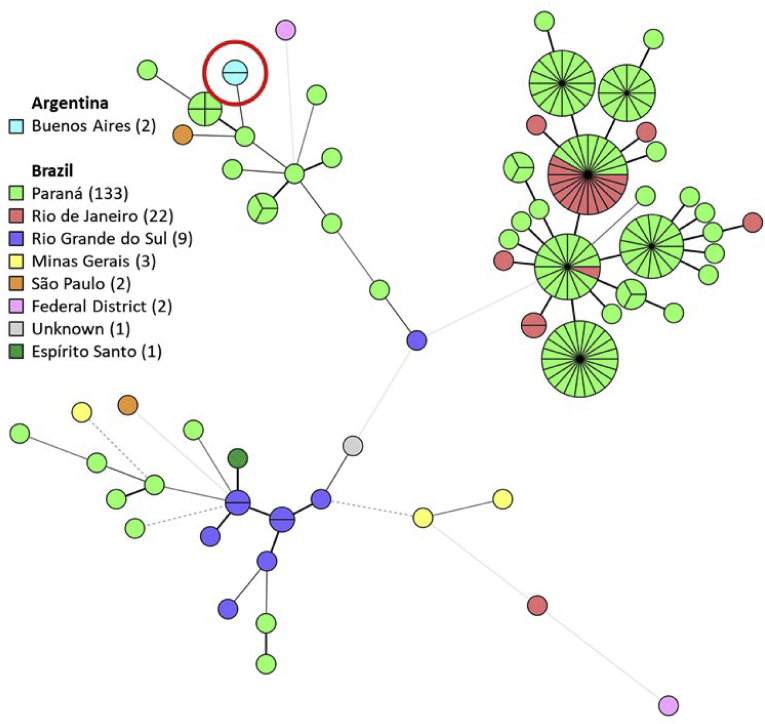
**Minimum-spanning tree of 175 clinical *Sporothrix brasiliensis* isolates.** Isolates are colored according to the Brazilian state of retrieval, or Buenos Aires, Argentina (red encircled light blue). Branches indicate the similarity between isolates with thick solid lines (variation in one allele), thin solid lines (variation in two alleles), thin dashed lines (variation in three alleles), and thin dotted lines (variation in four or more markers). Number of isolates per location is shown in the color keys. (For interpretation of the references to color in this figure legend, the reader is referred to the Web version of this article.)

Using STR genotyping, both *S. brasiliensis* isolates were found to display identical copy numbers for all nine markers, indicating a shared source. By comparing the current two isolates with historical data, the isolates were most related to isolates from Paraná state in the south of Brazil and differed in two markers (light green, Fig. 4). Isolates originating from São Paulo state were subsequently the most related (orange, Fig. 4).

## 3 Discussion

In Brazil, *S. brasiliensis* was first identified retrospectively in 1998 from feline and human clinical isolates from the metropolitan region of Rio de Janeiro, Brazil [9]. The WHO currently considers this mycosis as a Neglected Tropical Disease affecting the skin (NTD-S) which poses a major public health problem. Due to difficulties in controlling the chain of transmission from sick cats and clonal selection and expansion of strains during the current epidemic, this underscores the need for a One Health approach – i.e. a collaborative, multisectoral, and transdisciplinary approach for veterinary, public health and environmental sectors. In Brazil, during the period from 2011 to 2022, 13,475 cases of human sporotrichosis were reported in the metropolitan region of Rio de Janeiro and cases are currently reported in most states [10,11]. Between 2011 and 2014, the first report of zoonotic sporotrichosis in Argentina involved four cat-related cases of human sporotrichosis in Buenos Aires [12]. During the period 2010–2020, 54 cases of sporotrichosis were reported in Argentina, nine of which were identified as *S. brasiliensis* [13]. In retrospect, *S. brasiliensis* was first identified as a human pathogen in Buenos Aires in 1988 [14].

Among the human clinical manifestations of sporotrichosis, the lymphocutaneous form is most common reported in about 50–70% of cases [1]. This clinical form mainly affects the upper and lower extremities and the face. Lesions appear within days to weeks of infection as a papular skin lesion at the site of inoculation, which may progress to a nodule or ulcer. It is characterized by swelling, erythema and nodular lesions with erythematous halos, accompanied by itching. Subsequently, the lesions spread in a linear and step-like fashion along the regional lymphatic vessels towards the lymph nodes, i.e. in a sporotrichoid pattern. Nodular or fluctuant lesions tend to ulcerate [1].

Here, we report two cases of lymphocutaneous sporotrichosis affecting upper limbs. In case 1, the patient received SSKI for 16 weeks and in the second case the patient received itraconazole treatment for 12 weeks. In both cases the patients were treated as outpatient and did not attend for follow-up after the indicated periods of time.

In the clinical forms, lymphocutaneous or cutaneous, the recommended treatment (A-II) is itraconazole (200 mg per day orally) for 4 weeks, which can be extended depending on clinical resolution, and is associated with response rates of 80–100% [13,15]. For the disseminated cutaneous form, the dose of itraconazole can be increased to 400 mg/day in two doses. As an alternative treatment, SSKI is recommended (AII) in adults at 3–6 g/day orally, (initial dose of 5 drops, three times daily, and increased daily by 5 drops, up to a maximum dose of 30–40 drops), because of its bitter taste, it can be administered with milk or fruit juice. In sporotrichosis, whose pathogenesis involves the action of neutrophils, iodide is likely to be effective, through a mechanism of interaction with these cells, suppression of the production of toxic oxygen intermediates and consequent anti-inflammatory effect; in addition to halogenation reactions by myeloperoxidases, essential for phagocyte action [16]. SSKI has been used for the treatment of cutaneous sporotrichosis, with response rates between 70% and 89% [17]. Despite its efficacy, SSKI is less well tolerated than itraconazole, and its main side effects include gastritis, rhinitis, bronchitis, urticaria, erythema, metallic taste and salivary gland inflammation. The duration of antifungal therapy for the lymphocutaneous form is at least three months, but can be extended. It is advisable to continue treatment for another one to two months to avoid relapses. Some patients with mild forms of sporotrichosis and in pregnancy, especially the fixed cutaneous form, can be treated with local heat or thermotherapy. For those with severe sporotrichosis, a liposomal amphotericin B (5 mg/kg per day intravenously) is recommended.

Using STR typing, both isolates were found to have identical genotypes. It was previously found that STR typing correlates well with whole genome sequencing (WGS) single nucleotide polymorphism (SNP) analysis [2]. Therefore, these isolates likely originate from the same source, with cats as the most likely reservoir. Since the current found genotype is highly related to ones from Brazil, *S. brasiliensis* is likely introduced from Brazil to Argentina. This introduction might be facilitated by the movement of sick or colonized cats, as was recently demonstrated for the first cases of *S. brasiliensis* in the United Kingdom [4]. This hypothesis is further supported by the more recent emergence of the fungus when compared to Brazil [18]. Genotyping of more isolates from Argentina would be needed to determine whether all *S. brasiliensis* cases are imported or local introduction from the environment took place. Early diagnosis and timely treatment is necessary because it leads to a better prognosis of this emerging zoonosis.

## Conflicts of interest

None to disclose.

## Author contributions

Conceptualization: N.B.F,B.S,J.F.M. Methodology: N.B.F, B.S, sequencing: M.C.L.J; Formal Analysis genotyping: B.S,E.F.J.M; Investigation (clinical cases) N.B.F,I.N.T,A.L; Resources: N.B.F, B.S, E.F.J.M; Writing-Original draft: N.B.F,B.S; Writing- Review & Editing: N.B.F,B.S, E.F.J.M; Funding acquisition: E.F.J.M.

## References

[1] Queiroz-Telles F, Fernandez NB. Systemic Fungal Infection. Practical Manual. fourth ed. Asociación Panamericana de Infectologia. Sporotrichosis. Cap. 9 pag.147-160. Córdoba: Recfot, 2023. Digital Book. ISBN 978-987-4056-46-7[In Spanish].

[2] Spruijtenburg B., Bombassaro A., Meijer E.F.J., Rodrigues A.M., Grisolia M.E., Aparecida Vicente V. (2023). *Sporothrix brasiliensis* genotyping reveals numerous independent zoonotic introductions in Brazil. J. Infect..

[3] Barnacle J.R., Chow Y.J., Borman A.M., Wyllie S., Dominguez V., Russell K. (2023). The first three reported cases of *Sporothrix brasiliensis* cat-transmitted sporotrichosis outside South America. Medical Mycology Case Reports.

[4] Rossow J.A., Queiroz-Telles F., Caceres D.H., Beer K.D., Jackson B.R., Pereira J.G. (2020). A One health approach to combatting *Sporothrix brasiliensis*: narrative review of an emerging zoonotic fungal pathogen in South America. J Fungi (Basel).

[5] Thomson P., González C., Blank O., Ramírez V., Río C.d., Santibáñez S. (2023). Sporotrichosis outbreak due to *Sporothrix brasiliensis* in domestic cats in Magallanes, Chile: a One-Health-Approach Study. J. Fungi.

[6] do Prado C.M., Razzolini E., Santacruz G., Ojeda L., Geraldo M.R., Segovia N. (2023 Sep 27). First cases of feline sporotrichosis caused by *Sporothrix brasiliensis* in Paraguay. J Fungi (Basel).

[7] de Andrade Galliano Daros Bastos F., Raimundo Cognialli R.C., Rodrigues de Farias M., Dos Santos Monti F., Wu K. (2022). Spread of *Sporothrix* spp. through respiratory droplets from infected cats: a potential route of transmission. Med. Mycol..

[8] Fernandez N., Iachini R., Farias L., Pozzi N., Tiraboschi I. (2015). Proceedings of the Infocus 2015; 2015 Nov 5-7; Córdoba, Argentina.

[9] Marimon R., Cano J., Gené J., Sutton D.A., Kawasaki M., Guarro J. (2007 Oct). *Sporothrix brasiliensis, S. globosa, and S. mexicana,* three new Sporothrix species of clinical interest. J. Clin. Microbiol..

[10] Open WHO online Course Sporotrichosis: Training for national and district-level health workers. Module 5 Part 1: Brazil. Dayvison Saraiva Freitas INI-Fiocruz. Retrieved Dec 28, 2023 from: https://openwho.org/courses/NTDs-sporotrichosis.

[11] Bombassaro A., Spruijtenburg B., Medeiros F., Jacomel Favoreto de Souza Lima B., Ballardin L., Rodrigues de Farias M. (2023). Genotyping and antifungal susceptibility testing of *Sporothrix brasiliensis* isolates from Southern Brazil. Mycoses.

[12] Gremião I.D.F., Miranda L.H.M., Reis E.G., Rodrigues A.M., Pereira S.A. (2017). Zoonotic epidemic of sporotrichosis: cat to human transmission. PLoS Pathog..

[13] Santiso G., Messina F., Arechavala A., Marín E., Romero M.M., Sosa M.A. (2023). Esporotricosis en Argentina: análisis clínico y epidemiológico. Biomedica.

[14] Córdoba S., Isla G., Szusz W., Vivot W., Hevia A., Davel G., Canteros C.E. (2018 Jul). Molecular identification and susceptibility profile of *Sporothrix schenckii* sensu lato isolated in Argentina. Mycoses.

[15] Kauffman C.A., Bustamante B., Chapman S.W., Pappas P.G. (2007 Nov 15). Infectious diseases society of America. Clinical practice guidelines for the management of sporotrichosis: 2007 update by the infectious diseases society of America. Clin. Infect. Dis..

[16] Costa R.O., Macedo P.M., Carvalhal A., Bernardes-Engemann A.R. (2013 May-Jun). Use of potassium iodide in dermatology: updates on an old drug. An. Bras. Dermatol..

[17] Thompson G., Le T. (2021 December). Global guideline for the diagnosis and management of the endemic mycoses: an initiative of the European confederation of medical Mycology in cooperation with the international society for human and animal Mycology. Lancet Infect. Dis..

[18] Etchecopaz A., Toscanini M.A., Gisbert A., Mas J., Scarpa M., Iovannitti C.A. (2021). *Sporothrix brasiliensis*: a review of an emerging South American fungal pathogen, its related disease, presentation and spread in Argentina. Journal of Fungi.

